# A Prospective Study on the Progression, Recurrence, and Regression of Cervical Lesions: Assessing Various Screening Approaches

**DOI:** 10.3390/jcm13051368

**Published:** 2024-02-28

**Authors:** Tudor Gisca, Iulian-Valentin Munteanu, Ingrid-Andrada Vasilache, Alina-Sinziana Melinte-Popescu, Simona Volovat, Ioana-Sadyie Scripcariu, Raluca-Anca Balan, Ioana Pavaleanu, Razvan Socolov, Alexandru Carauleanu, Constantin Vaduva, Marian Melinte-Popescu, Ana-Maria Adam, Gigi Adam, Petronela Vicoveanu, Demetra Socolov

**Affiliations:** 1 Department of Mother and Child Care, “Grigore T. Popa” University of Medicine and Pharmacy Iasi, 700115 Iasi, Romaniaioana.scripcariu@umfiasi.ro (I.-S.S.); ioana-m-pavaleanu@umfiasi.ro (I.P.); razvan.socolov@umfiasi.ro (R.S.); petronela.pintilie@umfiasi.ro (P.V.); demetra.socolov@umfiasi.ro (D.S.); 2 Clinical and Surgical Department, Faculty of Medicine and Pharmacy, ‘Dunarea de Jos’ University, 800216 Galati, Romania; ana-maria.adam@ugal.ro; 3 Department of Mother and Newborn Care, Faculty of Medicine and Biological Sciences, ‘Ștefan cel Mare’ University, 720229 Suceava, Romania; alina.melinte@usm.ro; 4 Department of Medical Oncology, University of Medicine and Pharmacy ‘Grigore T Popa’, 700115 Iasi, Romania; 5 Department of Morphofunctional Sciences I, “Grigore T. Popa” University of Medicine and Pharmacy Iasi, 700115 Iasi, Romania; 6 Department of Mother and Child Medicine, Faculty of Medicine, University of Medicine and Pharmacy, 200349 Craiova, Romania; cristian.vaduva@umfcv.ro; 7 Department of Internal Medicine, Faculty of Medicine and Biological Sciences, ‘Ștefan cel Mare’ University, 720229 Suceava, Romania; melinte.marian@usm.ro; 8 Department of Pharmaceutical Sciences, Faculty of Medicine and Pharmacy, ‘Dunarea de Jos’ University, 800216 Galati, Romania

**Keywords:** cervical lesions, HPV genotyping, p16/Ki67 dual staining, cervical cytology, clinical risk factors

## Abstract

(1) Background: The prediction of cervical lesion evolution is a challenge for clinicians. This prospective study aimed to determine and compare the predictive accuracy of cytology, HPV genotyping, and p16/Ki67 dual staining alone or in combination with personal risk factors in the prediction of progression, regression, or persistence of cervical lesions in human papillomavirus (HPV)-infected patients; (2) Methods: This prospective study included HPV-positive patients with or without cervical lesions who underwent follow-up in a private clinic. We calculated the predictive performance of individual tests (cervical cytology, HPV genotyping, CINtecPlus results, and clinical risk factors) or their combination in the prediction of cervical lesion progression, regression, and persistence; (3) Results: The highest predictive performance for the progression of cervical lesions was achieved by a model comprising a Pap smear suggestive of high-grade squamous intraepithelial lesion (HSIL), the presence of 16/18 HPV strains, a positive p16/Ki67 dual staining result along with the presence of at least three clinical risk factors, which had a sensitivity (Se) of 74.42%, a specificity of 97.92%, an area under the receiver operating curve (AUC) of 0.961, and an accuracy of 90.65%. The prediction of cervical lesion regression or persistence was modest when using individual or combined tests; (4) Conclusions: Multiple testing or new biomarkers should be used to improve HPV-positive patient surveillance, especially for cervical lesion regression or persistence prediction.

## 1. Introduction

An accurate prediction of the progression, regression, or persistence of cervical lesions is critical for guiding appropriate clinical management decisions and ensuring optimal patient outcomes. While several screening methods, such as cytology and human papillomavirus (HPV) testing, have been effective in identifying individuals at risk, the development of more accurate predictive tools remains a priority.

The natural history of cervical lesions is influenced by HPV characteristics, such as the type, viral load, and persistence [1]. In addition to the viral characteristics, environmental or exogenous factors have been recognized as modifiers of the natural progression of HPV infections, which can lead to cervical cancer, and they include smoking, multiple pregnancies, prolonged use of hormonal contraceptives, and co-infection with sexually transmitted infections, such as *Chlamydia trachomatis* or human immunodeficiency virus (HIV) [2,3,4,5].

Depending on the institution, the results of a colposcopic biopsy may be reported as cervical intraepithelial neoplasia (CIN) 1, 2, or 3, according to the Richart histopathological grading system, or as histologic low-grade squamous intraepithelial lesion (LSIL) or high-grade squamous intraepithelial lesion (HSIL), according to the Lower Anogenital Squamous Terminology system [6,7]. As a general rule, histologic LSIL is equivalent to CIN1, while histologic HSIL is equivalent to CIN2 and CIN3. CIN3 is considered to be a more reliable diagnosis compared to CIN2, as expert pathologists show a remarkable agreement of over 80% on CIN3 diagnoses, while their agreement on CIN2 diagnoses is less than 30% [8]. CIN3 is also more likely to indicate a histological correlation with cellular transformation, carrying a significant risk of progression to cervical cancer. It is frequently linked to highly carcinogenic HPV genotypes [9].

Treatment is recommended for non-pregnant patients who have been diagnosed with CIN3 (HSIL) or adenocarcinoma in situ (AIS) [10]. On the other hand, patients with CIN2 can be referred to a specific treatment or can undergo further follow-up due to the risk of pre-term birth after excisional approaches, although this risk is questionable [11].

However, it is important to point out that CIN1 lesions have a natural history, which is marked by a high rate of spontaneous regression (approximately 80%) [12], but they can evolve to more severe cervical lesions (CIN2/3) in more than 10% of the cases [13]. A recent systematic review and meta-analysis conducted by Loopik et al., which included 89 studies, evaluated the regression, persistence, and progression rates of conservatively managed CIN 1-3 [14]. The results from this meta-analysis indicated that in women with conservatively treated CIN 1, the rates of regression, persistence, and progression to CIN 2-3 or worse were 60%, 25%, and 11%, respectively. For CIN 2, the overall rates of regression were 55%, persistence—23%, and progression—19%. Lastly, with regard to CIN 3, the corresponding percentages were 28%, 67%, and 2%. Less than 1% of women with CIN1 or 2 progressed to cervical cancer in this study.

Recent literature has outlined several tests, which could improve the detection and prognosis of cervical lesion progression. These tests include methylation assays of viral and host markers, immune cytodiagnosis, proteomic or transcriptomics panels [15,16,17,18]. The p16/Ki67 dual stain test (CINtecPlus) is a cutting-edge technology, which has been approved for the precise detection of cell transformation [19]. The presence of p16 and Ki67 indicates potential cellular transformation caused by HPV, as p16 demonstrates the disruption of the retinoblastoma pathway caused by E7 oncoproteins, while Ki67 serves as a marker of cellular proliferation [20]. Multiple studies have demonstrated that utilization of dual stain instead of Papanicolaou stain cytology can enhance the accuracy of identifying pre-cancerous conditions and distinguishing them from low-grade abnormalities in patients with positive HPV test results [20,21,22,23].

The risk stratification of progression, regression, or persistence of cervical lesions using the p16/Ki67 dual stain test was poorly studied in the literature. Moreover, many reports evaluated the predictive performance of the p16/Ki67 dual stain test in previously stained slides. Thus, the aim of this prospective study was to determine and compare the predictive accuracy of cytology, HPV genotyping, and p16/Ki67 dual staining (performed on freshly collected samples) taken individually or combined with personal risk factors in the progression, regression, or persistence of cervical lesions in patients with a proven HPV infection.

## 2. Materials and Methods

This prospective study was conducted at the Avicena Profertis Clinic in Iasi, Romania, between October 2022 and December 2023, and it included patients infected with HPV with or without cervical lesions who underwent follow-up or were programmed for excisional treatments (Ethical Approval No 728/01.10.2022). The inclusion criteria comprised the following: patients with HPV genotyping positive for at least one of the HPV strains who had indication for CINtecPlus testing and who gave their informed consent for participating in this study. The exclusion criteria consisted of patients with concomitant vaginal or vulvar pre-cancerous lesions, a negative HPV genotyping test, personal history of genital cancer, pregnancy, lack of informed consent.

In the first stage of the evaluation, patients underwent Pap testing and HPV genotyping. If the patients had a positive HPV genotyping result, they underwent CINtecPlus testing (Roche mtm Laboratories AG, Mannheim, Germany), and the results were recorded in the database.

**Figure 1:**
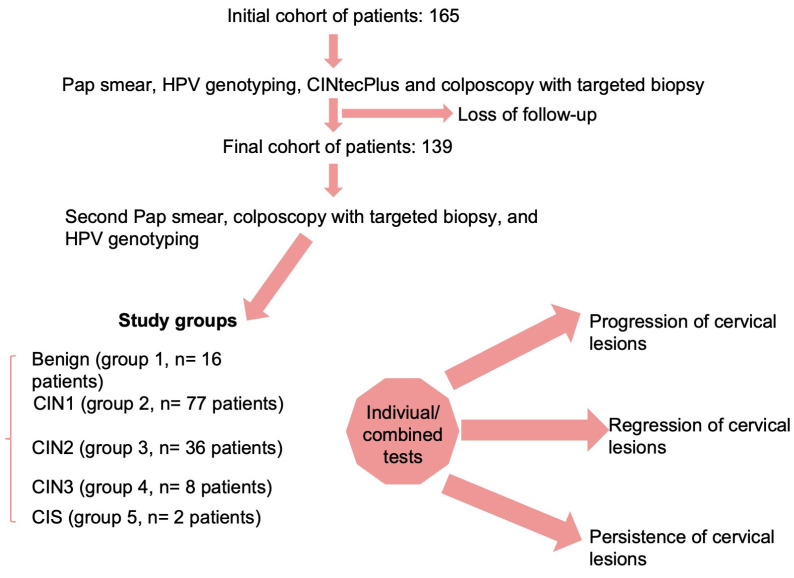
Diagram representing the study design.

Patients with abnormal Pap smear results, such as ASC-US, LSIL, and HSIL, underwent colposcopy examination and a targeted cervical biopsy of the suspected cervical lesions. Pathologists classified the histology findings on cervical biopsy or conization probes as either benign, CIN1, CIN2, CIN3, or microinvasive cervical cancer at the first examination and at the follow-up visit. The histopathological report was correlated with both cytology and HPV test results. Patients who had a histopathological diagnosis of CIN3 or microinvasive cervical cancer in this stage were excluded from the follow-up and underwent standard protocol, while patients with no documented histopathological lesions or with CIN1 or CIN2 were further included in the follow-up. These histology results were considered the gold standard. The initial cohort of patients comprised 165 subjects, but only 139 patients completed the follow-up program (Figure 1).

Patients were followed up in a one-year time frame, and during the second visit, they underwent a second Pap smear, colposcopy with targeted biopsy, and HPV genotyping. The results from these tests were also recorded.

Our database also included clinical data of the patients, such as age, BMI, clinical risk factors for cervical cancer, vaccination, and contraceptive history, as well as personal obstetrical and gynecological history.

We defined the progression of lesions in the following situations:

- if the initial histopathological diagnosis was CIN1 or CIN2, and at follow-up, the histopathological diagnosis was CIN2/CIN3 or microinvasive cervical cancer;
- if the initial histopathological diagnosis was CIN1 or CIN2, and at follow-up, the histopathological diagnosis was a combination of CIN1/ CIN2/CIN3 or microinvasive cervical cancer.

We defined a regression of lesions if the initial histopathological diagnosis was CIN1/CIN2, and at follow-up, the histopathological diagnosis was of a lower grade, such as negative/CIN1. The persistence of lesions was documented by the same histopathological result at follow-up.

Cases who tested negative for HPV or showed signs of new infections with different strains of HPV compared to baseline were considered to have a transitory HPV infection. In contrast, cases where the same genotype was discovered at follow-up were considered to have a persistent HPV infection.

The collected cervical samples were sent in a ThinPrep^®^ vial (Hologic BV, Da Vincilaan 5, 1930 Zaventem, Belgium) and processed on a ThinPrep 5000 (Hologic, Marlborough, MA, USA), stained using the Papanicolaou technique, microscopically observed by an experienced cytotechnician, and reviewed by the pathologists. The results were interpreted according to the Bethesda system from 2001, revised in 2014 [24], and they consisted of the following categories: negative for intraepithelial lesions or malignancy (NILM), atypical squamous cell—undetermined significance (ASC-US), atypical squamous cell—cannot exclude HSIL (ASC-H), low-grade intraepithelial lesion (LSIL), high-grade intraepithelial lesion (HSIL), atypical glandular cells (AGC), other.

HPV testing was performed on cervical samples using Allplex™ PCR System (Seegene Inc., Songpa-gu, Seoul, Republic of Korea) for detection of human papillomavirus-19 high-risk HPV types (16, 18, 26, 31, 33, 35, 39, 45, 51, 52, 53, 56, 58, 59, 66, 68, 69, 73, 82) and 9 low-risk HPV types (6, 11, 40, 42, 43, 44, 54, 61, 70).

Using the ThinPrep 5000 Processor (Hologic, Marlborough, MA, USA), a slide was produced retrospectively from the remaining cytology material for p16/Ki-67 immunostaining. Based on the instructions provided by the manufacturer, the CINtec PLUS Cytology kit (Roche mtm Laboratories AG, Mannheim, Germany) was used to assess the slides. If one cervical epithelial cell per slide was stained with both brown cytoplasmic (p16) and red nuclear (Ki-67) stain, the sample was categorized as positive; otherwise, it was termed negative if only one stain was visible. Some of the results from CINtecPlus testing are presented as Supplementary Materials (Supplementary Figures S1–S6).

Colposcopy was performed using a photo/video colposcope 3MLS LED 1”- LEISEGANG (Feinmechanik-Optik GmbH, Berlin, Germany). Colposcopists conducted the biopsies in accordance with established clinical protocol. As is standard procedure, hematoxylin and eosin (H&E) staining was applied to three sections of every biopsy sample.

Data description and univariate analysis comprised a chi-squared test for categorical variables, which were presented as frequencies with corresponding percentages, and an analysis of variance (ANOVA) with Bonferroni post hoc test for continuous variables, which were presented as means and standard deviations (SD). Univariate analysis was performed for the following groups according to the second histopathological results: benign (group 1, n = 16 patients), CIN1 (group 2, n = 77 patients), CIN2 (group 3, n = 36 patients), CIN3 (group 4, n = 8 patients), and in situ carcinoma (CIS) (group 5, n = 2 patients).

The primary outcomes were the progression, regression, and persistence of cervical lesions. We performed a sensitivity analysis using clinical risk factors for cervical cancer (smoking, immunosuppression, long-term use of oral contraceptives, multiple sexual partners, early debut of sexual activity, poor socioeconomic status, lack of HPV vaccination, family history of cervical cancer), the results from HPV genotyping, Pap smear, and CINtecPlus as predictors, taken individually or in combination, and considering the histopathological results of cervical biopsies as the gold standard. The statistical analyses were carried out using STATA SE (version 17, StataCorp LLC, College Station, TX, USA). A *p*-value of less than 0.05 was considered statistically significant.

## 3. Results

The database used for this analysis included 139 patients with HPV infections, who were segregated according to the second histopathological results into the following groups: benign (group 1, n = 16 patients), CIN1 (group 2, n = 77 patients), CIN2 (group 3, n = 36 patients), CIN3 (group 4, n = 8 patients), and in situ carcinoma (CIS) (group 5, n = 2 patients). Their demographic and clinical characteristics are presented in Table 1.

The mean age of patients who had CIN2, CIN3, or CIS was significantly higher compared to the age of patients with benign histopathological results or CIN1 (*p* = 0.0003). Moreover, the proportion of patients who had multiple sexual partners (>5) was significantly higher for CIN2, CIN3, and CIS groups compared to the other groups (*p* = 0.03).

Regarding Pap smear results, the first and second groups had a significantly higher proportion of NILM and ASC-US lesions, while the fourth and fifth groups presented with a significantly higher proportion of HSIL results. The second group also had the highest proportion of LSIL results, while the fifth group had a 50% rate of carcinoma based on the results of cervical cytology.

The results of HPV genotyping indicated that patients with CIN2, CIN3, and CIS had significantly higher rates of infection with high-risk HPV strains, such as HPV 16 and 18 (*p* < 0.001). On the other hand, we could not find any statistically significant difference between the groups regarding other high-risk (*p* = 0.05) or low-risk (*p* = 0.45) HPV strains.

A positive CINtecPlus result was significantly more frequently encountered among patients with CIN2, CIN3, and CIS (*p* < 0.001) compared to patients with a benign histopathological result or CIN1.

Finally, we could not find any statistically significant difference between the groups regarding clinical risk factors for cervical cancer, such as BMI, smoking status, early debut of sexual activity, prolonged use of oral contraceptives, a positive personal history of HPV vaccination, or invasive procedures on the cervix (*p* > 0.05).

In the second stage of our analysis, we evaluated the predictive accuracy of cytology, HPV genotyping, and p16/Ki67 dual staining (CINtecPlus) taken individually or combined with personal risk factors in the progression, regression, or persistence of cervical lesions in patients with a proven HPV infection.

Table 2 presents the predictive performance of all evaluated index tests and combined models considering the progression of cervical lesions (n = 34 patients, 24.46%). Our results indicated that the presence of at least three clinical risk factors (accuracy: 67.13%), a cervical cytology suggestive of HSIL (accuracy: 64.03%), the presence of 16/18 HPV strains (accuracy: 66.19%), and a positive CINtecPlus result (accuracy: 53.73%) had the highest performance in terms of accuracy.

**Figure 2:**
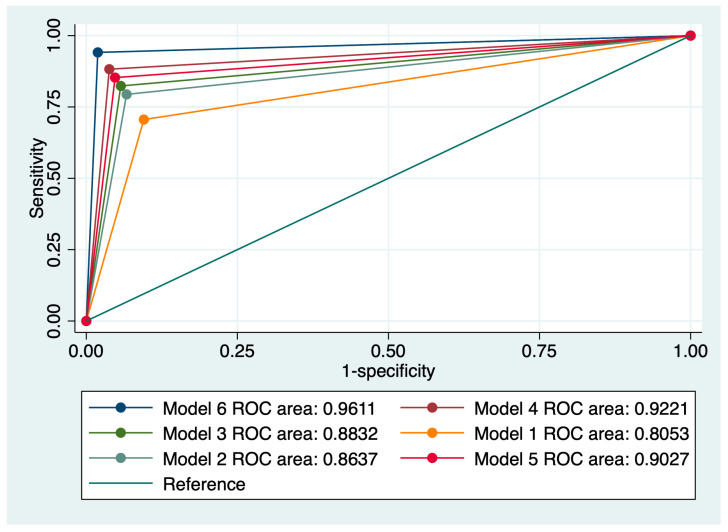
Comparison of ROC curves corresponding to six models used for the prediction of cervical anomaly progression.

We also evaluated various combinations of index tests, which resulted in six combined models. The highest predictive performance for the progression of cervical lesions was achieved by a model comprising a Pap smear suggestive of HSIL, the presence of 16/18 HPV strains, a positive CINtecPlus result along with the presence of at least three clinical risk factors (Model 6). This model was characterized by a sensitivity (Se) of 74.42%, a specificity of 97.92%, an area under the receiver operating curve (AUC) of 0.961, and an accuracy of 90.65%. Figure 2 outlines a comparison of all evaluated models taking into account the value of the ROC curve.

Table 3 presents the predictive performance of all evaluated index tests and combined models considering the regression of cervical lesions (n = 19 patients, 13.66%). The presence of low-risk HPV strains (accuracy: 73.72%), a Pap smear suggestive of ASC-US (accuracy: 66.19%), a negative CINtecPlus result (accuracy: 40.3%), and a personal history of fewer than three risk factors for cervical cancer (accuracy: 64.03%) best predicted the regression of cervical abnormalities.

**Figure 3:**
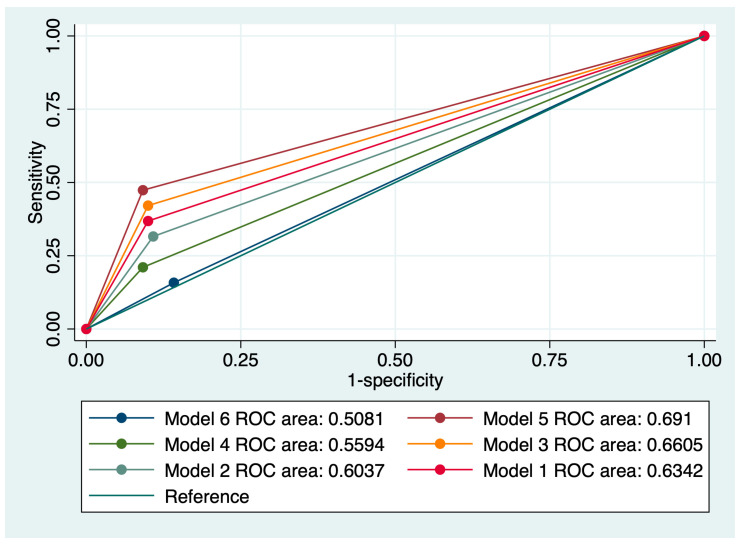
Comparison of ROC curves corresponding to six models used for the prediction of cervical anomaly regression.

Additionally, a model comprising a cervical cytology suggestive of LSIL, in the absence of HPV 16/18, a negative CINtecPlus result, and a personal history of fewer than three risk factors for cervical cancer (Model 5) achieved the best results in terms of predicting the regression of cervical abnormalities (Se: 33.3%, Sp: 88%, AUC: 0.691, and accuracy: 82.14%). Figure 3 outlines a comparison of all evaluated models taking into account the value of the ROC curve.

**Figure 4:**
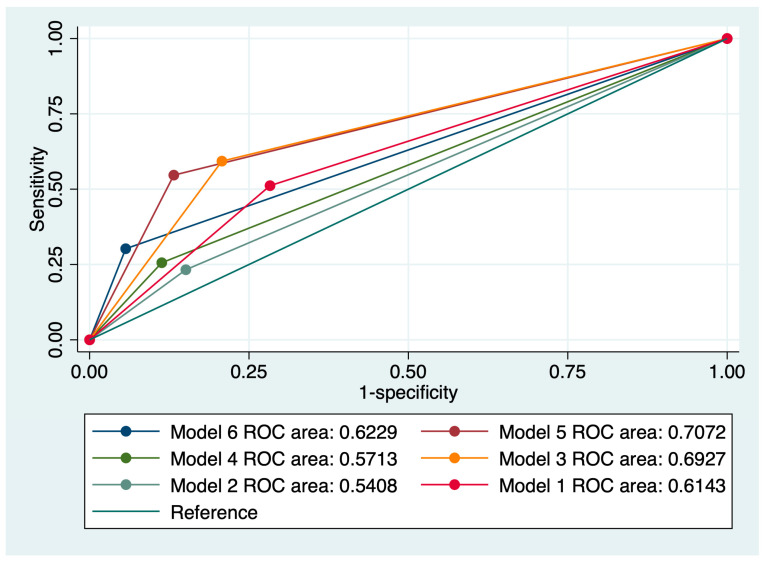
Comparison of ROC curves corresponding to six models used for the prediction of cervical anomaly persistence.

The final analysis evaluated the persistence of cervical abnormalities, and the results are presented in Table 4 and Figure 4. The overall accuracy of individual index tests in the prediction of cervical abnormality persistence was low to moderate—between 38.13 and 58.27%—the latter accuracy being attributed to a cervical cytology suggestive of LSIL. Moreover, all models that included LSIL outperformed models that included HSIL when used in the prediction of cervical abnormality persistence. The best predictive performance for this outcome was achieved by Model 5, which comprised a cervical cytology suggestive of LSIL, the presence of HPV 16/18 strains, a positive CINtecPlus result, and at least three clinical risk factors for cervical cancer, with a sensitivity of 61.04%, a specificity of 88.71%, an AUC value of 0.707, and an accuracy of 73.38%.

## 4. Discussion

The risk stratification of cervical abnormalities represents a challenge for clinicians, especially for patients with CIN2, who have not completed their family planning due to the controversial risk of pre-term birth after excisional therapies. On the other hand, it is important to note that the follow-up of patients with cervical abnormalities relies on tests characterized by variable sensitivities and specificities depending on the studied population.

This study was concentrated on HPV-positive patients who underwent follow-up during a one-year time frame, and we evaluated the predictive accuracy of various index tests (cytology, HPV genotyping, and CINtecPlus) taken individually or combined with clinical risk factors in the progression, regression, or persistence of cervical abnormalities.

Our results indicated that the progression of cervical abnormalities was most accurately predicted by the presence of at least three clinical risk factors (accuracy: 67.13%), a cervical cytology suggestive of HSIL (accuracy: 64.03%), the presence of 16/18 HPV strains (accuracy: 66.19%), and a positive CINtecPlus result (accuracy: 53.73%). Most of the evaluated index tests had high specificity and low-to-moderate sensitivity. A model, which reunited all these individual index tests, achieved a Se of 74.42%, a specificity of 97.92%, an AUC of 0.961, and an accuracy of 90.65%.

Indeed, a high-grade index cytology, the presence of 16/18 HPV strains, and a positive CINtecPlus result have been associated with a higher risk of progression of cervical lesions [25,26,27]. The comparative literature data regarding the accuracy of the combined model in the prediction of cervical lesion progression is missing, but there are several studies, which investigated the predictive performance of various combinations of index tests in disease detection. Thus, in a recent observational prospective study, the authors found out that a combination of p16/Ki67 dual staining and HPV 16/18 had a sensitivity of 42.8% and a specificity of 92.8% in the detection of high-grade CIN2 lesions [28]. Moreover, the same authors outlined a sensitivity of 44% and a specificity of 91% in the detection of high-grade CIN3 lesions.

When we evaluated the performance of individual index tests in the prediction of cervical abnormality regression, we found out that the presence of low-risk HPV strains (accuracy: 73.72%), a Pap smear suggestive of ASC-US (accuracy: 66.19%), a negative CINtecPlus result (accuracy: 40.3%), and a personal history of fewer than three risk factors for cervical cancer (accuracy: 64.03%) achieved the best results. Follow-up studies confirmed a higher rate of cervical lesion regression in the presence of low-grade lesions (ASC-US/LSIL), infection with HPV other than HPV-16, or a negative CINtecPlus result [29,30]. Our model, which reunited all these index tests, achieved the best results in the prediction of cervical lesion regression (Se: 33.3%, Sp: 88%, AUC: 0.691, and accuracy: 82.14%).

Finally, a model comprising a cervical cytology suggestive of LSIL, the presence of HPV 16/18 strains, a positive CINtecPlus result, and at least three clinical risk factors for cervical cancer achieved a Se of 61.04%, a Sp of 88.71%, an AUC value of 0.707, and an accuracy of 73.38% in the prediction of cervical lesion persistence.

The prediction of cervical lesion regression or persistence was modest in terms of sensitivity and overall accuracy, even though it included factors associated with regression or persistence of cervical lesions [31,32], thus leaving treatment algorithms dependent on repeated examinations and testing. Moreover, these results indicate the need for inclusion of more sensitive tests, which can outperform the classical approaches. This finding is supported by recent studies, which outline a higher predictive performance of various molecular or methylation markers [33,34].

Louvanto et al. investigated the predictive performance of pyrosequencing methylation and HPV genotyping in the prediction of regression, persistence, or progression of HSIL (CIN2) in a 2-year surveillance period [34]. The authors demonstrated that the S5 classifier outperformed cytology and HPV genotyping when used for the prediction of regression versus progression of cervical lesions. Moreover, a combination of the S5 classifier and cytology had an AUC value of 0.735 in comparing regression versus progression of cervical lesions, whereas HPV genotyping did not provide additional prognostic benefit.

Several risk factors for the recurrence of cervical lesions have been proposed in the literature [35]. One study conducted by Bogani et al. investigated the impact of persistent HPV infection on the recurrence risk of CIN2 + in a cohort of 545 patients who underwent primary conization [36]. Their results indicated that patients with persistent HPV infection after 6 months had a risk of recurrence of 7.46%, while patients with persistent HPV infection at 12 months after conization had a risk of cervical lesion recurrence of 13.1%. On the other hand, they found out that the persistence of HPV infection for more than 12 months did significantly increase the risk of recurrence. These results point out the need to thoroughly follow up patients with persistent HPV infection in order to timely detect cervical lesion recurrence after primary conization.

The limitations of this study are represented by the small cohort of patients included in the follow-up, the limited time frame, and inclusion of patients with a cytology positive for only ASC-US, LSIL, or HSIL. We hypothesize that a longer follow-up period would allow us to better understand the dynamics of cervical lesion progression, regression, or persistence, especially in patients with a high-risk profile. The strengths of this study stem from the use of histopathology results as the gold standard both at inclusion in the study and at follow-up, the assessment of the predictive accuracy of various models in the prediction of progression, regression, and persistence of cervical lesions, as well as a CINtecPlus assay performed on fresh cytology samples. We advocate for the use of CINtecPlus assay on fresh cytology samples and not on already colored slides because this approach could offer better insight into the markers’ positivity and limit the false-negative results. Moreover, this study had a prospective design, and it was conducted during a one-year time frame. We will follow up these patients for another year and report the results in another paper.

Our results outline the limited performance of current individual screening assays in the prediction of cervical lesion regression or persistence, thus pointing out the need for performing multiple tests during the patient’s follow-up. Our results clearly indicate that the combined screening approaches have superior predictive performance in comparison with individual screening tests in the prediction of cervical lesion progression, persistence, or regression. Thus, clinicians should integrate the data obtained from multiple screening tests and corroborate them with literature data, which indicate a high or a low risk of cervical lesion progression, regression, or persistence. Moreover, we hypothesize that the inclusion of new biomarkers could improve the overall prognostic accuracy and reduce the long-term costs of the HPV-positive patient surveillance program. Additionally, deep neural networks could be employed for improvement of cervical lesion detection and classification [37,38].

Finally, this study indicated low HPV vaccinal rates and raised awareness with regard to the need to implement national policies, which will increase the take-up of HPV vaccination, which is low in Romania [39].

## 5. Conclusions

Cervical lesion progression was best predicted by a model, which included the presence of at least three clinical risk factors, a cervical cytology suggestive of HSIL, the presence of 16/18 HPV strains, and a positive CINtecPlus result.

The prediction of cervical lesion regression or persistence was modest using individual index tests or combined models, thus outlining the need to improve the current surveillance of HPV-positive patients either via multiple testing or by including new biomarkers.

Further follow-up studies on various populations of HPV-positive patients could offer us a better insight into specific prediction models.

## Figures and Tables

**Table 1:** Demographic and clinical characteristics segregated according to the second histopathological examination of the cervical probes.

Variable	Benign (Group 1, n = 16 Patients)	CIN1 (Group 2, n = 77 Patients)	CIN2 (Group 3, n = 36 Patients)	CIN3 (Group 4, n = 8 Patients)	CIS (Group 5, n = 2 Patients)	*p*-Value
Age, years (mean ± SD)	27.5 ± 4.78	34.90 ± 9.75	37.25 ± 9.69	39.5 ± 6.36	48.77 ± 13.81	0.0003
BMI, kg/m^2^ (mean ± SD)	24.02 ± 3.80	24.04 ± 5.04	23.63 ± 4.57	29.37 ± 4.70	22.67 ± 5.73	0.22
Smoking (n/%)	Yes = 1 (6.25%)	Yes = 4 (5.19%)	Yes = 3 (8.33%)	Yes = 1 (12.5%)	Yes = 1 (50%)	0.17
Multiple sexual partners (n/%)	Yes = 1 (6.25%)	Yes = 2 (2.60%)	Yes = 6 (16.67%)	Yes = 2 (25%)	Yes = 1 (50%)	0.03
Early debut of sexual activity (n/%)	Yes = 0 (0%)	Yes = 7 (9.09%)	Yes = 4 (11.11%)	Yes = 1 (12.5%)	Yes = 0 (0%)	0.70
Prolonged use of oral contraceptives (n/%)	Yes = 1 (6.25%)	Yes = 11 (14.29%)	Yes = 8 (22.22%)	Yes = 1 (12.5%)	Yes = 1 (50%)	0.19
Personal history of HPV vaccination (n/%)	Yes = 0 (0%)	Yes = 3 (3.89%)	Yes = 3 (8.33%)	Yes = 1 (12.5%)	Yes = 0 (0%)	0.26
Personal history of conization/LLETZ (n/%)	Yes = 0 (0%)	Yes = 11 (14.29%)	Yes = 8 (22.22%)	Yes = 1 (12.5%)	Yes = 1 (50%)	0.19
Cervical cytology (n/%)	NILM = 5 (31.25%) ASC-US = 9 (56.25%) LSIL = 2 (12.5%) HSIL = 0 (0%) Carcinoma = 0 (0%)	NILM = 3 (3.90%) ASC-US = 31 (40.26%) LSIL = 37 (48.05%) HSIL = 6 (7.79%) Carcinoma = 0 (0%)	NILM = 0 (0%) ASC-US = 6 (16.67%) LSIL = 14 (38.89%) HSIL = 16 (44.44%) Carcinoma = 0 (0%)	NILM = 0 (0%) ASC-US = 2 (25%) LSIL = 1 (12.50%) HSIL = 5 (62.5%) Carcinoma = 0 (0%)	NILM = 0 (0%) ASC-US = 0 (0%) LSIL = 0 (0%) HSIL = 1 (50%) Carcinoma = 1 (50%)	<0.001
HPV 16/18 (n/%)	Yes = 1 (6.25%)	Yes = 15 (19.48%)	Yes = 18 (50%)	Yes = 7 (87.50%)	Yes = 2 (100%)	<0.001
Other high-risk HPV strains (n/%)	Yes = 12 (80%)	Yes = 37 (48.05%)	Yes = 22 (61.11%)	Yes = 3 (37.50%)	Yes = 0 (0%)	0.05
Low-risk HPV strains (n/%)	Yes = 2 (12.50%)	Yes = 16 (21.05%)	Yes = 3 (8.57%)	Yes = 2 (25.00%)	Yes = 0 (0%)	0.45
CINtecPlus results (n/%)	Positive = 3 (20.00%)	Positive = 31 (41.33%)	Positive = 30 (83.33%)	Positive = 5 (83.33%)	Positive = 2 (100%)	<0.001

Table legend: CIN—cervical intraepithelial neoplasia; CIS—in situ carcinoma; SD—standard deviation; BMI—body mass index; HPV—human papillomavirus; LLETZ—large loop excision of the transformation zone; NILM—negative for intraepithelial neoplasia; ASC-US—atypical squamous cell—undetermined significance; LSIL—low-grade intraepithelial lesion; HSIL—high-grade intraepithelial lesion.

**Table 2:** Predictive performance of various index tests and models in the prediction of cervical abnormality progression.

Index Test	Se (%)	Sp (%)	NPV (%)	AUC	Accuracy
Cervical cytology, ASC-US	41.18	44.16	62.96	0.5440	43.24
Cervical cytology, LSIL	32.3	59.05	72.94	0.4570	52.5
Cervical cytology, HSIL	17.65	79.05	74.47	0.4835	64.03
HPV 16/18	44.12	73.33	80.21	0.4127	66.19
Other high-risk HPV	64.71	50	81.25	0.4265	53.62
Low-risk HPV	11.76	81.55	73.68	0.5334	64.23
Positive CINtecPlus	63.64	50.5	80.95	0.4293	53.73
Clinical risk factors (at least three)	44.12	74.31	81	0.5873	67.13
Cytology, LSIL + HPV 16/18 (Model 1)	51	89.13	78.1	0.805	76.26
Cytology, HSIL + HPV 16/18 (Model 2)	60	92.55	82.86	0.863	82
Cytology, LSIL + HPV 16/18 + Positive CINtecPlus (Model 3)	65.12	93.75	85.71	0.883	84.89
Cytology, HSIL + HPV 16/18 + Positive CINtecPlus (Model 4)	69.77	95.83	87.62	0.922	87.7
Cytology, LSIL + HPV 16/18 + Positive CINtecPlus + Clinical risk factors (Model 5)	70.7	94.9	88.55	0.902	87.7
Cytology, HSIL + HPV 16/18 + Positive CINtecPlus + Clinical risk factors (Model 6)	74.42	97.92	89.52	0.961	90.65

Legend: Se—sensitivity; Sp—specificity; NPV—negative predictive value; AUC—area under the curve; HPV—human papillomavirus; ASC-US—atypical squamous cell—undetermined significance; LSIL—low-grade intraepithelial lesion; HSIL—high-grade intraepithelial lesion.

**Table 3:** Predictive performance of various index tests and models in the prediction of cervical abnormality regression.

Index Test	Se (%)	Sp (%)	NPV (%)	AUC	Accuracy
Cervical cytology, ASC-US	52.63	68.33	90.11	0.604	66.19
Cervical cytology, LSIL	10.53	56.66	80	0.336	50.36
Cervical cytology, HSIL	21.05	80	86.49	0.505	71.94
HPV 16/18	10.53	65.83	82.29	0.618	58.27
Other high-risk HPV	72.2	49.17	92.19	0.618	52.17
Low-risk HPV	15.79	83.05	85.96	0.505	73.72
Negative CINtecPlus	26.32	42.61	77.7	0.655	40.3
Clinical risk factors (fewer than three)	31.58	69.17	86.4	0.503	64.03
Cytology, LSIL + HPV 16/18 (Model 1)	23.3	88.99	80.83	0.634	74.8
Cytology, HSIL + HPV 16/18 (Model 2)	20.69	88.29	80.99	0.603	74.29
Cytology, LSIL + Negative HPV 16/18 + Negative CINtecPlus (Model 3)	32	89.57	85.71	0.660	79.29
Cytology, HSIL + HPV 16/18 + Negative CINtecPlus (Model 4)	26.27	87.20	90.83	0.559	80.71
Cytology, LSIL + Negative HPV 16/18 + Negative CINtecPlus + Clinical risk factors (fewer than three) (Model 5)	33.3	88	91.67	0.691	82.14
Cytology, HSIL + Negative HPV 16/18 + Negative CINtecPlus + Clinical risk factors (fewer than three) (Model 6)	14.29	85.71	85	0.508	75

Legend: Se—sensitivity; Sp—specificity; NPV—negative predictive value; AUC—area under the curve; HPV—human papillomavirus; ASC-US—atypical squamous cell—undetermined significance; LSIL—low-grade intraepithelial lesion; HSIL—high-grade intraepithelial lesion.

**Table 4:** Predictive performance of various index tests and models in the prediction of cervical abnormality persistence.

Index Test	Se (%)	Sp (%)	NPV (%)	AUC	Accuracy
Cervical cytology, ASC-US	27.91	54.72	31.87	0.413	38.13
Cervical cytology, LSIL	47.67	75.47	47.06	0.615	58.27
Cervical cytology, HSIL	20.93	81.13	38.74	0.510	43.88
HPV 16/18	30.23	67.92	37.50	0.509	44.60
Other high-risk HPV	45.35	32.69	26.56	0.500	40.58
Low-risk HPV	19.05	86.79	40.35	0.470	45.26
Positive CINtecPlus	54.88	50.00	41.27	0.475	52.99
Clinical risk factors (more than three)	25.58	60.38	33.33	0.503	38.85
Cytology, LSIL + HPV 16/18 (Model 1)	47.83	78.72	43.53	0.614	58.27
Cytology, HSIL + HPV 16/18 (Model 2)	25.64	86.89	47.75	0.540	52.5
Cytology, LSIL + HPV 16/18 + Positive CINtecPlus (Model 3)	55.70	83.33	58.82	0.692	67.63
Cytology, HSIL + HPV 16/18 + Positive CINtecPlus (Model 4)	28.21	90.16	49.55	0.571	55.40
Cytology, LSIL + HPV 16/18 + Positive CINtecPlus + Clinical risk factors (more than three) (Model 5)	61.04	88.71	64.71	0.707	73.38
Cytology, HSIL + HPV 16/18 + Positive CINtecPlus + Clinical risk factors (more than three) (Model 6)	34.21	95.24	54.55	0.622	61.87

Legend: Se—sensitivity; Sp—specificity; NPV—negative predictive value; AUC—area under the curve; HPV—human papillomavirus; ASC-US—atypical squamous cell—undetermined significance; LSIL—low-grade intraepithelial lesion; HSIL—high-grade intraepithelial lesion.

## Data Availability

The datasets are available from the corresponding authors upon reasonable request due to local policies.

## Supplementary Materials

The following supporting information can be downloaded at: https://www.mdpi.com/article/10.3390/jcm13051368/s1, Figure S1: CINtec plus positive. Co-expression of p16 (brown) signals and Ki-67 (red) signals in the same atypical squamous cells (×20); Figure S2: CINtec plus negative. Expression of Ki-67 (red) signals in atypical squamous cells (×40); Figure S3: CINtec plus negative. No expression of Ki-67 and p16 in squamous cells (×20); Figure S4: CINtec plus positive. Co-expression of p16 (brown) signal (weak) and Ki-67 (red) signal in the same squamous cells (×20); Figure S5: CINtec plus positive. Co-expression of p16 and Ki-67 in the same atypical squamous cells (×20); Figure S6: CINtec plus positive. Co-expression of p16 and Ki-67 in the same atypical squamous cell (×40).
