# Supplementary figures and images for: A Prospective Study on the Progression, Recurrence, and Regression of Cervical Lesions: Assessing Various Screening Approaches
Source: J Clin Med. 2024 Feb 28;13(5):1368. doi: 10.3390/jcm13051368 (PMC10931951; doi:10.3390/jcm13051368)

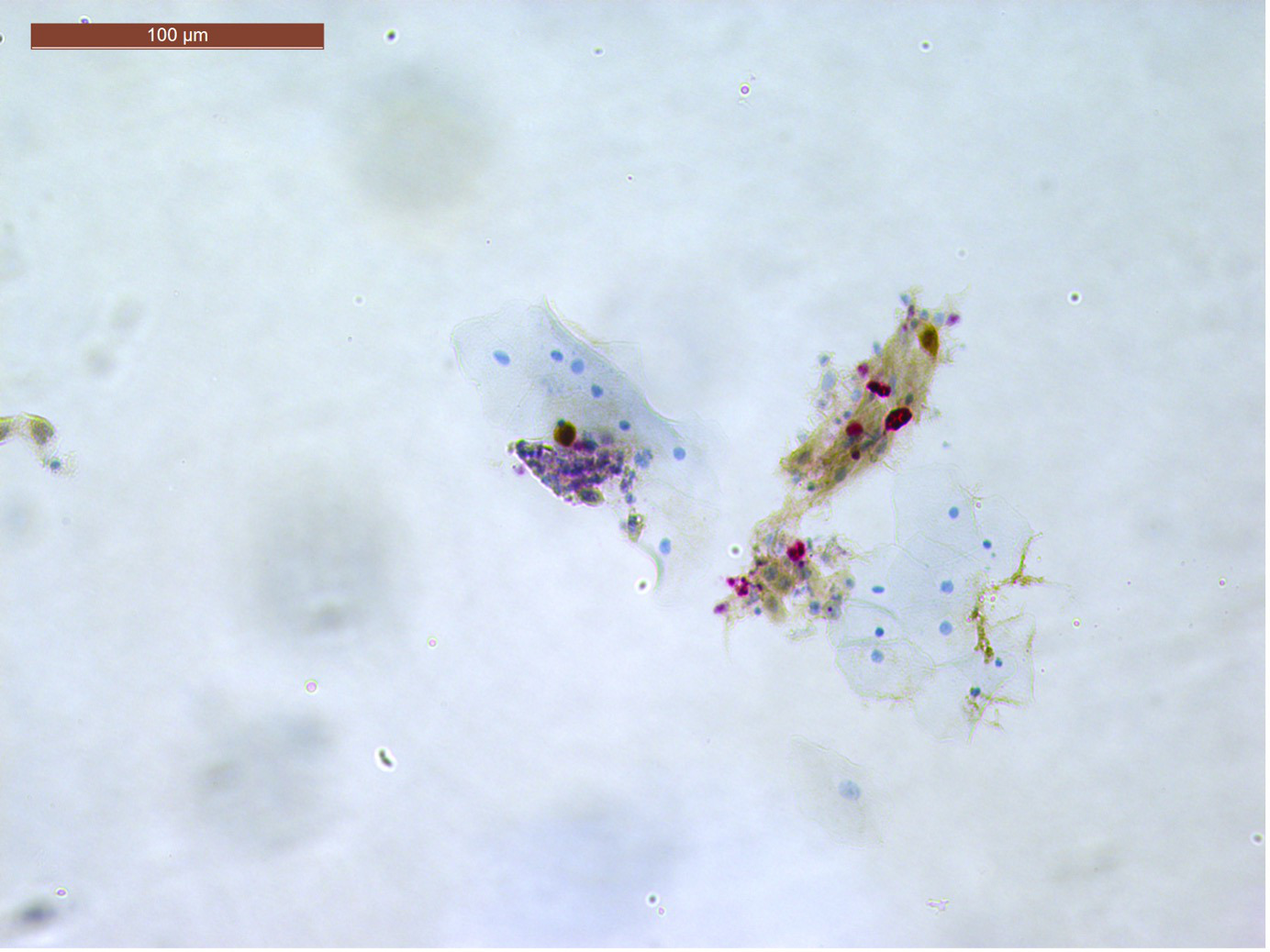

Supplement: Supplementary file 1 [file jcm-13-01368-s001.zip › jcm-2866890-supplementary/S1.jpg]

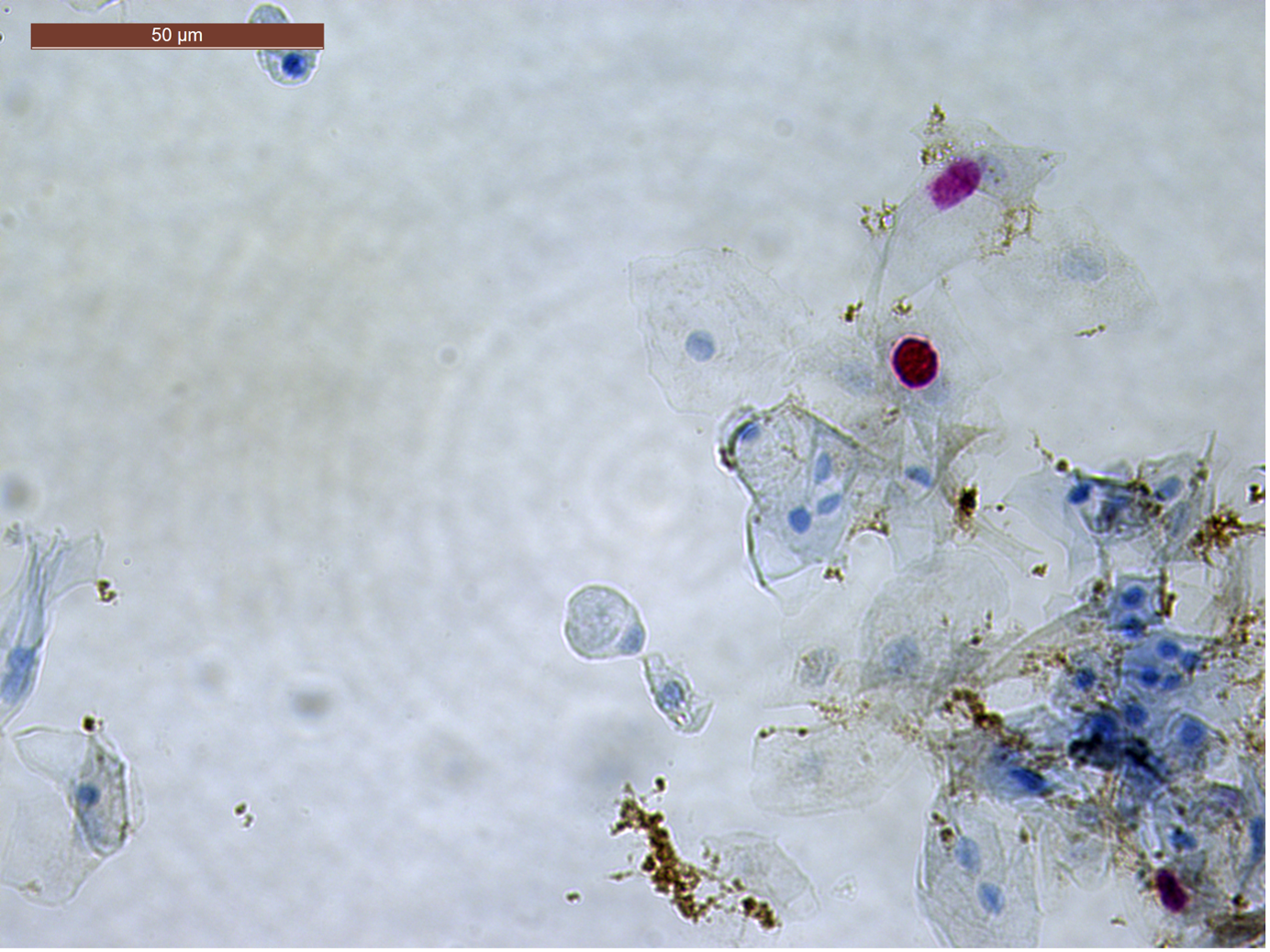

Supplement: Supplementary file 1 [file jcm-13-01368-s001.zip › jcm-2866890-supplementary/S2.png]

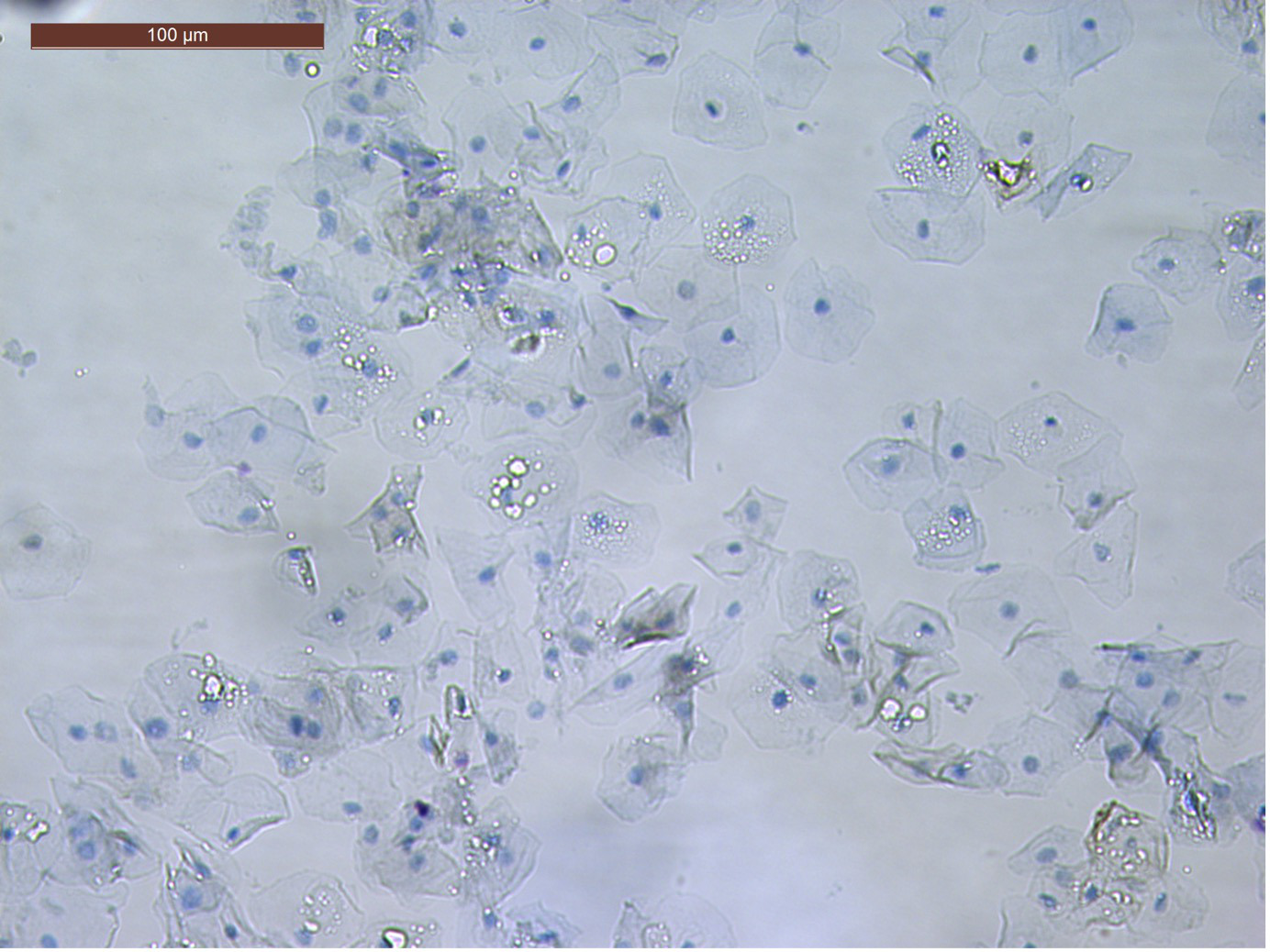

Supplement: Supplementary file 1 [file jcm-13-01368-s001.zip › jcm-2866890-supplementary/S3.jpg]

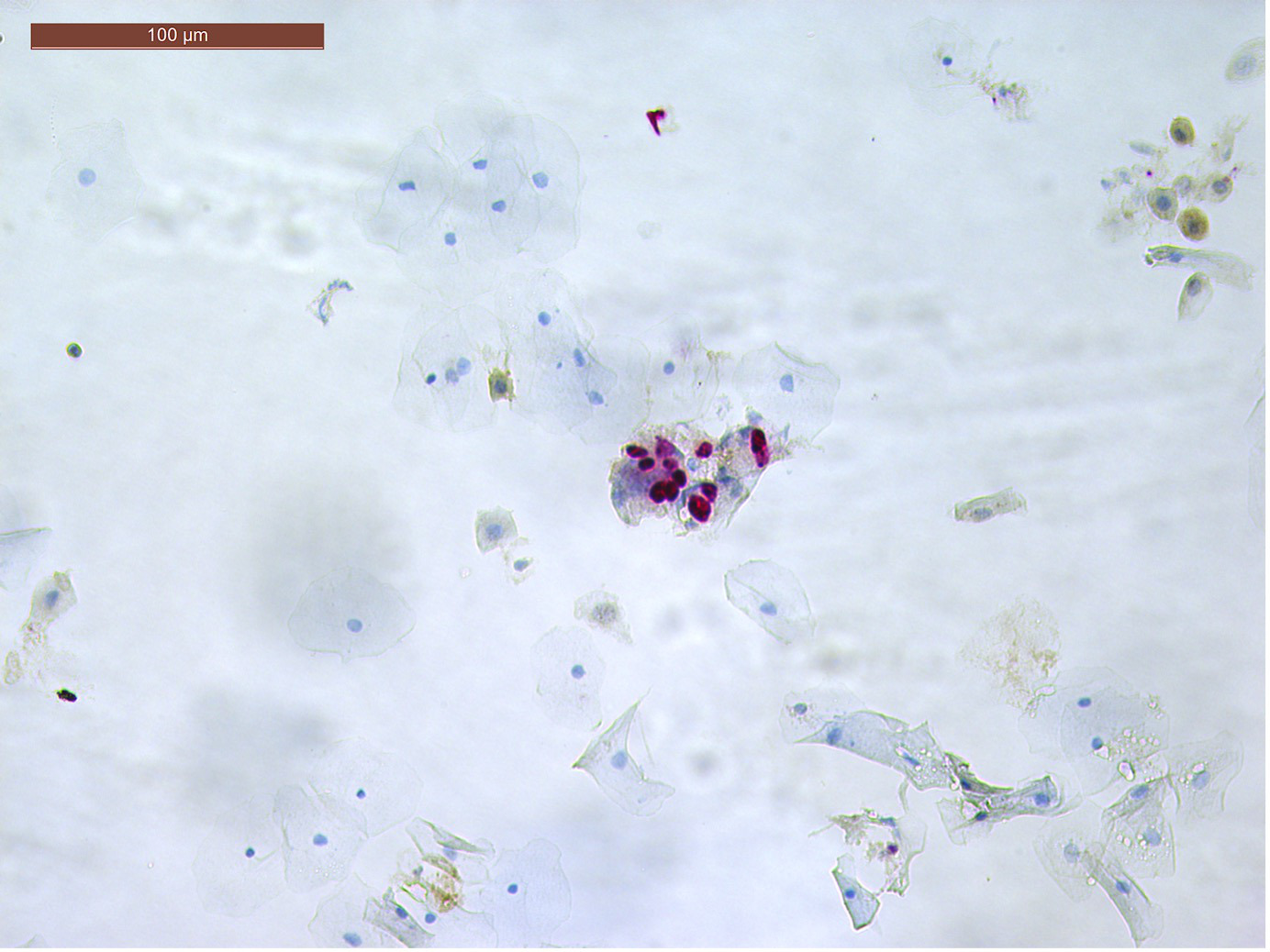

Supplement: Supplementary file 1 [file jcm-13-01368-s001.zip › jcm-2866890-supplementary/S4.jpg]

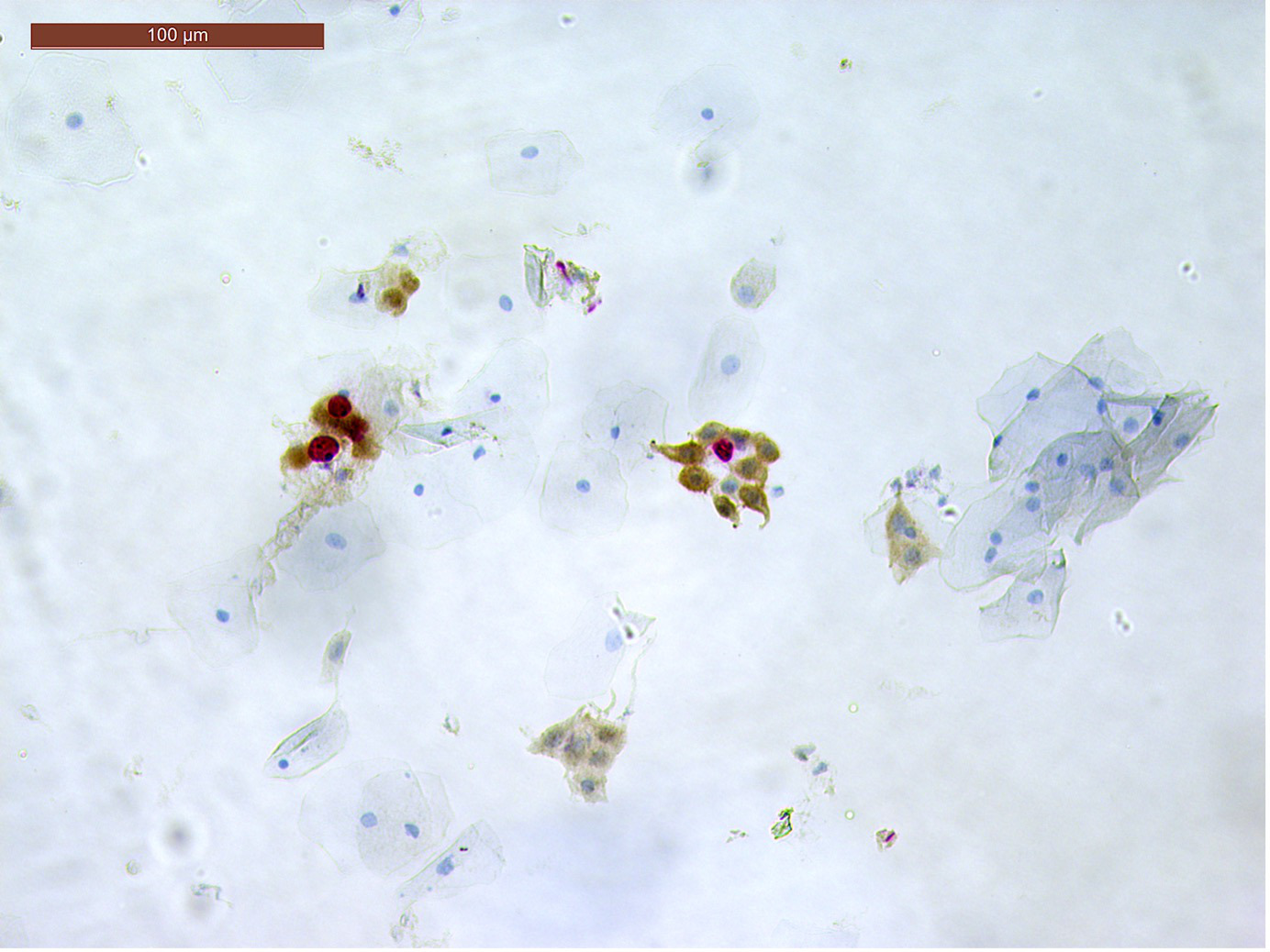

Supplement: Supplementary file 1 [file jcm-13-01368-s001.zip › jcm-2866890-supplementary/S5.jpg]

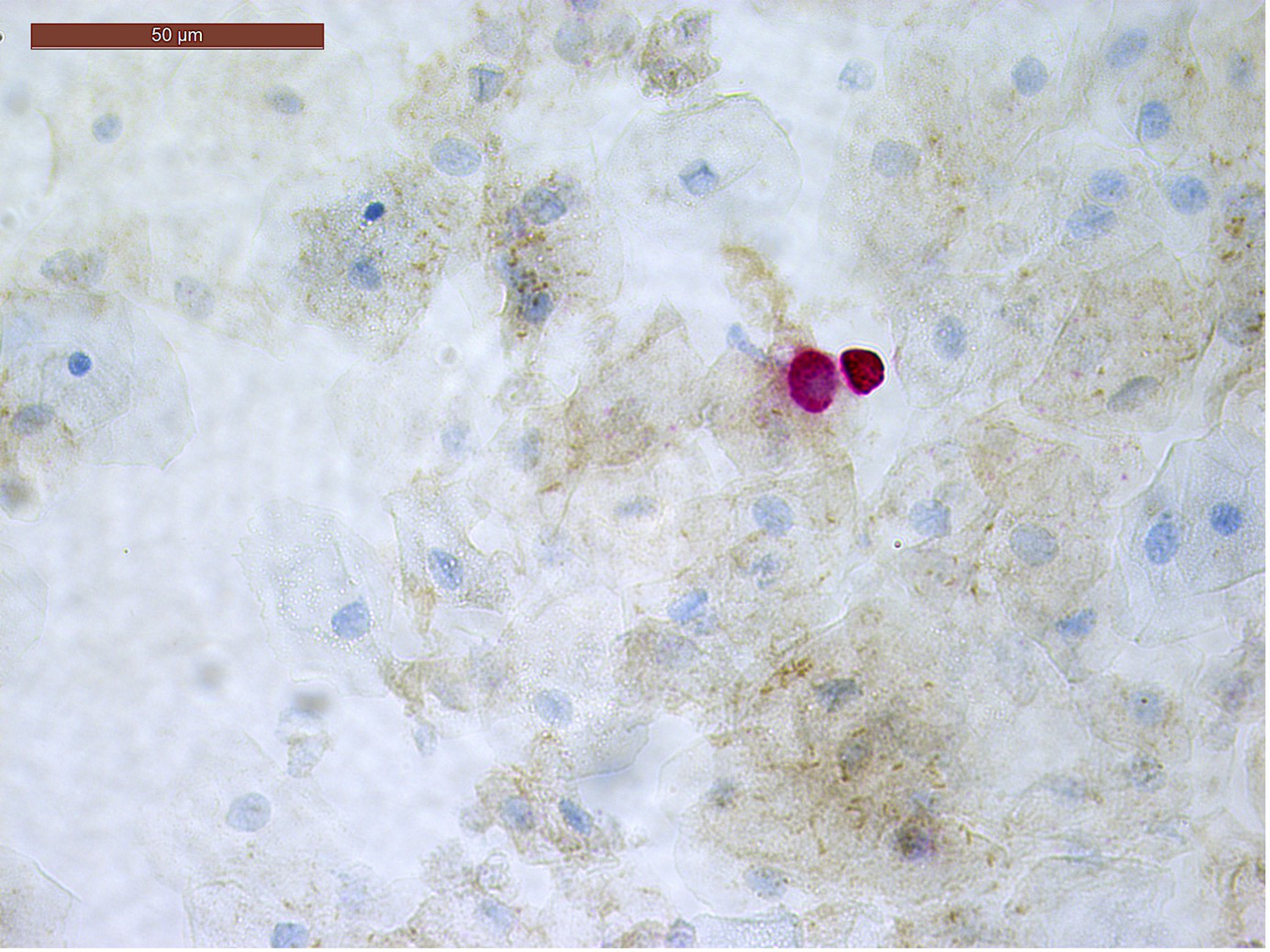

Supplement: Supplementary file 1 [file jcm-13-01368-s001.zip › jcm-2866890-supplementary/S6.jpg]
